# Physical activity differentially regulates VEGF, PlGF, and their receptors in the human placenta

**DOI:** 10.14814/phy2.14710

**Published:** 2021-01-19

**Authors:** Jayonta Bhattacharjee, Shuhiba Mohammad, Alexandra D. Goudreau, Kristi B. Adamo

**Affiliations:** ^1^ School of Human Kinetics Faculty of Health Sciences University of Ottawa Ottawa ON Canada

**Keywords:** angiogenesis, physical activity, placenta, pregnancy

## Abstract

Physical activity (PA) has beneficial effects on the function of many organs by modulating their vascular development. Regular PA during pregnancy is associated with favorable short‐ and long‐term outcomes for both mother and fetus. During pregnancy, appropriate vascularization of the placenta is crucial for adequate maternal–fetal nutrient and gas exchange. How PA modulates angiogenic factors, VEGF, and its receptors in the human placenta, is as of yet, unknown. We objectively measured the PA of women at 24–28 and 34–38 weeks of gestation. Participants were considered “active” if they had met or exceeded 150 min of moderate‐intensity PA per week during their 2nd trimester. Term placenta tissues were collected from active (*n* = 23) or inactive (*n* = 22) women immediately after delivery. We examined the expression of the angiogenic factors VEGF, PlGF, VEGFR‐1, and VEGFR‐2 in the placenta. Western blot analysis showed VEGF and its receptor, VEGFR‐1 was significantly (*p* < 0.05) higher both at the protein and mRNA levels in placenta from physically active compared to inactive women. No difference in VEGFR‐2 was observed. Furthermore, immunohistochemistry showed differential staining patterns of VEGF and its receptors in placental endothelial, stromal, and trophoblast cells and in the syncytial brush border. In comparison, PlGF expression did not differ either at the protein or mRNA level in the placenta from physically active or inactive women. The expression and localization pattern of VEGF and its receptors suggest that PA during pregnancy may support a pro‐angiogenic *milieu* to the placental vascular network.

## Graphical abstract

We aimed to assess the expression of key angiogenic factors of the VEGF family in the term placenta of women based on physical activity status. The expression of VEGF and VEGFR‐1 was higher in placental samples from active vs. inactive women.

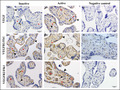

## 1 INTRODUCTION

Pregnancy is characterized by considerable physiological changes in hemodynamics, including increased resting heart rate and cardiac output, decreased peripheral vascular resistance, and dynamic changes in the placental vasculature (Sierra‐Laguado et al., 2006). Rapid fetal growth during pregnancy is accompanied by a 40–50% increase in maternal blood volume (Cid & Gonzalez, 2016; Sibai & Frangieh, 1995). These changes lead to increased blood flow throughout the placenta and increased maternal–fetal nutrient and gaseous exchange. The placenta is a highly vascular organ, and its blood flow continuously increases as pregnancy progresses, aided by proper vasculogenesis and angiogenesis. Placental vasculogenesis and angiogenesis underlie many critical physiological processes, including embryogenesis, as well as guiding appropriate placenta development and function. The imbalance between pro‐ and anti‐angiogenic factors is implicated in the etiology of preeclampsia and intrauterine growth restriction (Hoffmann et al., 2017; Tun et al., 2019). Therefore, maintaining a properly functioning placenta is of crucial importance for a healthy and successful pregnancy.

It is well‐established that physical activity (PA) during pregnancy benefits the whole maternal–fetal unit, not only during pregnancy but across the lifespan of both mother and offspring (Ferraro et al., 2012). Evidence indicates that maternal PA is preventive for the development of preeclampsia (Genest et al., 2012), gestational hypertension (Fortner et al., 2011), and gestational diabetes mellitus (Davenport et al., 2018; Han et al., 2012). Therefore, it is logical that placental function could also benefit from the effects of PA during pregnancy. Few studies demonstrate that PA during pregnancy improves placental growth rate, villous vascular volume, and functional capacity by increasing terminal villi proliferation (Bergmann et al., 2004; Clapp, 2006; Clapp et al., 2000). The mechanism(s) through which PA generates these effects on the human placenta has not been elucidated. As the placenta is the only organ through which maternal–fetal nutrient and gaseous exchange occur, the positive effect is likely to occur through the placenta's blood supply (Wang, 2010). However, a thorough examination of the relationships between maternal PA and the human placental vasculature and its developmental regulation has not been conducted.

Maintenance and growth of the placental vasculature are primarily sustained by vascular endothelial growth factor (VEGF), placenta growth factor (PlGF), and their receptors, VEGFR‐1 and VEGFR‐2 (Ahmed et al., 2000). Interactions between VEGF/PlGF and their receptors are the key functional determinants of angiogenesis and/or vasculogenesis. The functions of these factors are mainly mediated by binding with its two receptors; VEGF receptor‐1 (VEGFR‐1/FLT1) and ‐2 (VEGFR‐2/FLK1/KDR). VEGF also regulates vasodilation, vascular permeability, vascular protection, and endothelial cell survival (Ferrara et al., 2003). Therefore, VEGF and its receptor proteins should regulate any modifications or adaptations of the placental vasculature. While the VEGF protein family is fundamentally involved in normal placental function by modulating vascular growth, data on the VEGF family of proteins in healthy human placenta in the context of habitual maternal PA are missing. The present study aimed to determine the influence of PA during pregnancy on the expression and localization of VEGF, PlGF, and VEGFRs in the human placenta at term.

## 2 MATERIALS AND METHODS

### 2.1 Participant recruitment and ethics statement

Forty‐five healthy pregnant women were recruited from the local Ottawa region (ON, Canada) as part of the PhysicaL ACtivity and diEtary implicatioNs Throughout pregnAncy (PLACENTA) study. Participants were screened by specific inclusion and exclusion criteria of the PLACENTA study (summarized in Table 1). This study was approved by the University of Ottawa Research Ethics Board (file number: H11‐15‐29) and the Ottawa Health Science Network Research Ethics Board (OHSN‐REB), formerly the Ottawa Hospital Research Ethics Board (protocol number: 20160178‐01H), and was performed by following the ethical standards of the Declaration of Helsinki and its later amendments. All participants gave their informed written consent before inclusion in the study.

**TABLE 1:** Participant inclusion and exclusion criteria.

Inclusion criteria	Exclusion criteria
Maternal age of 18–40 years. Self‐reported pre‐pregnancy body‐mass index (BMI) of 18.5–29.9 kg/m^2^. Singleton pregnancy of less than 28 weeks gestation. Weight stable (±5 kg) for at least 6 months prior to pregnancy. No contraindications to exercise.	Pre‐pregnancy insulin‐treated diabetes. Diagnosis of fetal growth restriction or hypertensive diseases. Untreated thyroid disease. Hypertension requiring medication. Planning to have the child adopted. Unable to communicate in English or French.

### 2.2 Participant visits and accelerometer data capture

All participants visited the laboratory twice during their pregnancy from 24 to 28 weeks, and 34 to38 weeks of gestation. Maternal PA was assessed objectively using accelerometry, allowing for higher validity and reliability of PA measures (Colley et al., 2011). It is imperative to quantify PA using objective measures as our group has reported gross overestimations when using data obtained from self‐reported PA questionnaires (Brett et al., 2015). Participants were provided with an omniaxial Actical® accelerometer (Philips Respironics, OR, USA) and instructed to wear it for seven days following each laboratory visit. Bouts of free‐living PA were recorded during this time. A minimum of 10 h of wear time constituted a valid wear day, and a minimum of 4 valid wear days was required for data analysis (Colley et al., 2010). Data were then downloaded and analyzed by SAS version 9.4 (SAS Institute, NC, USA) following the protocols used by the Canadian Health Measures Survey (Colley et al., 2010). Cutoffs for accelerometer count ranges were as follows: <100 counts per minute (cpm) for sedentary time, 100 to less than 1535 cpm for light‐intensity PA (LPA), 1535 to less than 3962 for moderate‐intensity PA (MPA), and 3962 or more for vigorous‐intensity PA (VPA) (Colley & Tremblay, 2011). The evidence‐based Canadian guideline for PA throughout pregnancy recommends that pregnant women should participate in at least 150 min of moderate PA per week (Mottola et al., 2018). Participants were considered to have an “Active” PA status if they met or exceeded 150 min (or 21.4 min per day) of moderate‐intensity PA or greater (moderate‐to‐vigorous PA; MVPA) during mid‐pregnancy (24–28 weeks gestation). Conversely, those participants who were well below 150 min per week of moderate PA during mid‐pregnancy were considered to have an “Inactive” PA status. PA data from the late‐pregnancy study period (34–38 weeks gestation) were assessed but not used to define PA status as habitual activity patterns change as birth approaches. An Automated Self‐Administered 24‐h (ASA24) Dietary Assessment Tool, version ASA24‐Canada‐2018 (National Cancer Institute, Bethesda, MD) was used for dietary data collection and analysis during the study periods. A minimum of 3 days (2 weekdays and 1 weekend day) of intake data were collected as is required for analysis. Infant morphometric measurements, including height and weight, and skinfold thickness measurements were performed within 24–48 h after delivery following standard procedures (CDC, 2016; Schmelzle & Fusch, 2002). Fetal:placental birthweight ratio was calculated by dividing measured fetal weight by trimmed placental weight. Birthweight percentiles were determined using Canadian standards (Kramer et al., 2001). Furthermore, Infant weight‐for‐length z‐scores were calculated using the World Health Organization standard protocols (WHO, 2006).

### 2.3 Tissue sampling and preservation

Placental sampling was completed as described previously (Burton et al., 2014) with some modifications. Term placental tissue was collected immediately after vaginal delivery or via cesarean section. Placental weight was recorded after the removal of the umbilical cord and fetal membranes. All sampling was conducted promptly on ice, and areas of necrosis or calcification were avoided. Tissue from the chorionic and basal (decidua) plates was also avoided. To ensure adequate representation of heterogeneous placenta tissue sampling, tissues from three central and two peripheral cotyledons were selected. The central and peripheral portions of the tissues were pooled (2:1 ratio, respectively) and snap‐frozen immediately in liquid nitrogen. Then, two full‐thickness biopsies were taken from the periumbilical region, at 2–3 cm distance of the umbilical cord insertion for histological analysis. The tissue biopsies were fixed in 10% buffered formalin for 48 h at room temperature, then processed and embedded in paraffin by standard methods. Snap frozen tissues were stored at −80°C for protein extraction and RNA isolation.

### 2.4 Western blot analysis

Western blot was performed as described previously (Bhattacharjee et al., 2010). Briefly, frozen term placenta tissues from both the placenta of active (*n* = 23) and inactive (*n* = 22) women were homogenized and total protein was extracted in Radioimmunoprecipitation assay (RIPA) lysis buffer (50 mM Tris–HCl, pH 7.5; 150 mM NaCl; 1% v/v Triton X‐100; 1% w/v sodium deoxycholate; 0.1% w/v SDS; 1 mM Na_2_VO_3_; 25 mM NaF) with protease inhibitor cocktail (MilliporeSigma, Oakville, ON, Canada). Homogenized tissues were centrifuged at 15,000 *g* at 4°C for 15 min and supernatants were collected for total protein. Protein concentrations were determined using a DC protein assay (Bio‐Rad Laboratories, ON, Canada). An equal amount of total protein (50 µg) from each sample was subjected to SDS‐PAGE electrophoresis in reducing conditions on a 4–15% mini‐protean TGX precast gel (Bio‐Rad Laboratories) at 120 V for 60 min. Electrophoresis of separated proteins was transferred to a PVDF membrane (Bio‐Rad Laboratories) by electroblotting at 100 V for 60–90 min at room temperature. Blots were then blocked in 7% non‐fat dry milk in TBS with 0.1% TWEEN^®^20 (TBST) for 60 min at room temperature. After blocking, blots were incubated with mouse anti‐human monoclonal VEGF (Abcam Inc, ON, Canada), PlGF (Santa Cruz Biotechnology, CA, USA), VEGFR‐1 (Abcam), and VEGFR‐2 (Santa Cruz) primary antibodies (summarized in Table 2) in 5% non‐fat dried milk in TBST overnight at 4°C. Thereafter, the blots were washed three times in TBST and incubated for 60 min at room temperature with goat anti‐mouse secondary antibody labeled with HRP Conjugate (Bio‐Rad Laboratories) diluted in 5% milk in TBST. After washing three times with TBST, the peroxidase‐labeled blots were incubated with Clarity Western ECL Substrate (Bio‐Rad Laboratories) and visualized using a ChemiDoc XRS Machine (Bio‐Rad Laboratories). The blots were probed with beta‐actin antibody (Sigma) for normalization. The protein bands were quantified using Bio‐Rad's Image Lab software (Version 6.0.1; Bio‐Rad Laboratories).

**TABLE 2:** Antibodies used in the study.

Protein target	Name of antibody	Manufacturer (Catalog no., Lot no., RRID:)	Species raised in; monoclonal or polyclonal	Dilution, techniques & relevant references
VEGFA	Anti‐VEGF antibody (EP1176Y)	Abcam (Cat no. ab52917, Lot no. GR3219705‐8, RRID: AB_883427)	Rabbit polyclonal	1:10,000 in Western blot (Wang et al., 2020)
VEGFA	VEGF antibody (VG1)	Novus Biologicals (Cat no. NB100‐664, Lot no. E‐4, RRID: AB_10001947)	Mouse monoclonal	1:100 in Immunohistochemistry (O'Byrne et al., 2000; Turley et al., 1998)
PlGF	PlGF (H‐4)	Santa Cruz (Cat no. sc‐518003, Lot no. G0318, RRID: AB_2861376)	Mouse monoclonal	1:1000 in Western blot (Zhao et al., 2019)
VEGFR1	Anti‐VEGF receptor 1 antibody [Flt‐1/EWC]	Abcam (Cat no. ab9540, Lot no. GR3237737‐2, RRID: AB_307328)	Mouse monoclonal	1:1000 in Western blot (Lin et al., 2020)
VEGFR1	Anti‐VEGF receptor 1 antibody [Y103]	Abcam (Cat no. ab32152, Lot no. GR157619‐56, RRID: AB_778798)	Rabbit monoclonal	1:100 in Immunohistochemistry (Astern et al., 2013)
VEGFR2	Flk‐1 (A‐3)	Santa Cruz (Cat no. sc‐6251, Lot no. C1119, RRID: AB_628431)	Mouse monoclonal	1:500 in Western blot (Bersini et al., 2018; Francis & Wei, 2010); 1:50 in Immunohistochemistry (Möller et al., 2005)
Actin	Monoclonal anti‐β‐actin	Sigma (Cat no. A2228, Lot no. 067M4856V, RRID: AB_476697)	Mouse monoclonal	1:10,000 in Western blot (Mpilla et al., 2019; Palme et al., 2020)
Secondary	Goat anti‐mouse IgG	Bio‐Rad Laboratories (Cat no. 170‐6516, RRID: AB_11125547)	Goat	1:10,000 in Western blot
Secondary	Goat anti‐mouse whole IgG affinity‐purified antibodies	Jackson ImmunoResearch Laboratories (Cat no. 115‐065‐003, Lot no. 139869, RRID: AB_2338557)	Goat	1:500 in Immunohistochemistry
Secondary	Goat anti‐rabbit whole IgG affinity‐purified antibodies	Jackson ImmunoResearch Laboratories (Cat no. 111‐065‐003, Lot no. 140103, RRID: AB_2337959)	Goat	1:500 in Immunohistochemistry

### 2.5 RNA isolation and quantitative real‐time polymerase chain reaction (qPCR)

Total RNA was isolated from snap‐frozen placenta samples using an illustra RNAspin Mini Kit (GE Healthcare Life Sciences, ON, Canada) following the manufacturer's instructions. Isolated RNA eluted in RNase‐free water was assessed for quality and quantity by spectrophotometry (Gene5; BioTek Instruments, Inc., VT, USA). The integrity of the RNA was confirmed by a 2% agarose gel stained with SYBR Safe DNA gel stain (Invitrogen, CA, USA). One microgram of total RNA was reverse transcribed into cDNA using 5X iScript Reverse Transcription Supermix (Bio‐Rad Laboratories). The cDNA samples were then amplified by one‐step fast real‐time quantitative Polymerase Chain Reaction (qPCR) using a Roto‐Gene RG‐3000 (Corbett Research, Australia) detection system. We analyzed the expression of VEGF, PlGF, VEGFR‐1, and VEGFR‐2 genes deemed relevant candidates of the VEGF pathway of angiogenesis. In all placental samples, GAPDH was used as the endogenous control. All qPCR reactions were performed in duplicate. TaqMan gene expression assay probes of *GAPDH* (Hs02786624_g1), *VEGF* (Hs00900055_m1), *PLGF* (Hs00182176_m1), *VEGFR1* (Hs01052961_m1) and *VEGFR2* (Hs00911700_m1) were purchased from Applied Biosystems (CA, USA). The threshold cycle (CT) values of all samples were recorded, and relative gene expression was analyzed by 2^−ΔΔCT^ methods (Livak & Schmittgen, 2001). Gene expression values from physically inactive women were considered as control, and the relative gene expression of physically active women was determined. The data were normalized using the ratio of the target genes (*VEGF*, *PLGF*, *VEGFR*‐*1* or *VEGFR*‐*2*) to that of the endogenous control, *GAPDH*.

### 2.6 Immunohistochemistry

Paraffin‐embedded placental tissues were cut at 4 µm thickness and mounted on Superfrost Plus slides (Fisher Scientific, ON, Canada). Immunohistochemistry was performed as described previously (Bhattacharjee et al., 2010). Briefly, tissue sections were deparaffinized and rehydrated, and then washed in Tris‐buffered saline with 0.05% TWEEN^®^20 (TBST). Thereafter, heat‐mediated antigen retrieval was performed using sodium citrate buffer (10 mM, pH 6.0). The slides were then incubated with 10% normal goat serum (MilliporeSigma) for 60 min at room temperature. The slides were incubated overnight at 4°C with anti‐human monoclonal antibodies to VEGF (Novus Biologicals, CO, USA), VEGFR‐1 (Abcam), and VEGFR‐2 (Santa Cruz) (summarized in Table 2). Then the slides were incubated with appropriate biotinylated secondary antibodies (Jackson ImmunoResearch Laboratories, Inc., PA, USA) for 60 min at room temperature followed by a 30 min incubation with ExtraAvidin peroxidase (MilliporeSigma). The reaction was revealed using diaminobenzidine (DAB) chromogen (Abcam). The sections were counterstained using Harris' hematoxylin (Electron Microscopy Sciences, PA, USA), and then mounted with the aqueous mounting medium, Entellan^®^ (Merck KGaA, Darmstadt, Germany). Images were captured with a Zeiss Axio Imager M2 upright epifluorescent microscope equipped with Zen Blue version 2.3 (Carl Zeiss Microscopy GmbH, Köln, Germany). Negative controls were performed for each antigen by substituting the primary antibody with diluent only (TBST).

### 2.7 Statistical analysis

All data are shown as mean ± SD. The qPCR data were first analyzed by the gene expression analysis software Rotor‐Gene 6 (version 6.1; Corbett Research). All statistical analyses were conducted using GraphPad Prism Software (version 8.3.4; GraphPad Software Inc., CA, USA). Normality was assessed using the Shapiro–Wilk test. Anthropometric, western blot, and qRT‐PCR data were statistically analyzed by a Student's *t*‐test or Mann–Whitney *U* test where appropriate. A Student's *t*‐test was also used to compare changes in protein/gene expression in those women who remained “active” (i.e., ≥21.4 min of MVPA/day or meeting Guidelines) versus those who decreased their PA levels, thus no longer meeting the criteria for “active” status during the 3rd trimester. A two‐way mixed ANOVA with Bonferroni's multiple comparisons posttest was used to examine the effect of offspring sex on protein/gene expression. Differences were considered significant when *p* < 0.05.

## 3 RESULTS

### 3.1 Study participants demographics, physical activity data, and clinical characteristics

According to accelerometer data capture, 23 participants met or exceeded recommendations outlined by the Canadian guidelines and achieved, on average a minimum of 21.4 min/day of MPA or greater (MVPA) during the 2nd‐trimester window and were thus categorized as physically active. A total of 22 participants did not meet these criteria and were classified as inactive. Of those who were active during the 2nd trimester, *n* = 21 accelerometry data points were available for analysis. A total of 52% (*n* = 11) of participants in the active group maintained PA levels at the end of their 3rd trimester (supplemental Figure 1). Participant demographics including age, height, pre‐pregnancy weight, and BMI, gestational age at birth, mode of delivery, offspring sex, placental weight, offspring birth weight, length, birthweight percentile, body fat percentage, and weight‐for‐length z‐scores, and maternal PA data are shown in Table 3. There were no significant differences between active and inactive participants for age, pre‐pregnancy weight/BMI, and gestational age at birth (Table 3). During the 2nd trimester, there was a significant difference in the minutes of MPA, MVPA, and VPA in active versus inactive pregnant women (*p* < 0.0001) with no differences in LPA (Table 3). At the end of the 3rd trimester, women categorized as active based on the 2nd trimester data achieved significantly higher levels of MPA and MVPA, but not VPA or LPA compared to those defined as inactive during the 2nd trimester. Diet intake data revealed no differences between groups with‐respect‐to total caloric intake during either study period, but active women consumed greater amounts of carbohydrates and fiber in the 2nd trimester compared to inactive women (Table 3). In the 3rd trimester, active women consumed significantly more fiber than inactive women, with no differences in any other macronutrient measured (Table 3). While within the normal range (Wallace et al., 2013), comparably, the weights of the placenta from physically active women were reduced when compared to those from inactive women (*p* < 0.05), while there was no difference in the fetal:placental weight ratio (Table 3).

**FIGURE 1:**
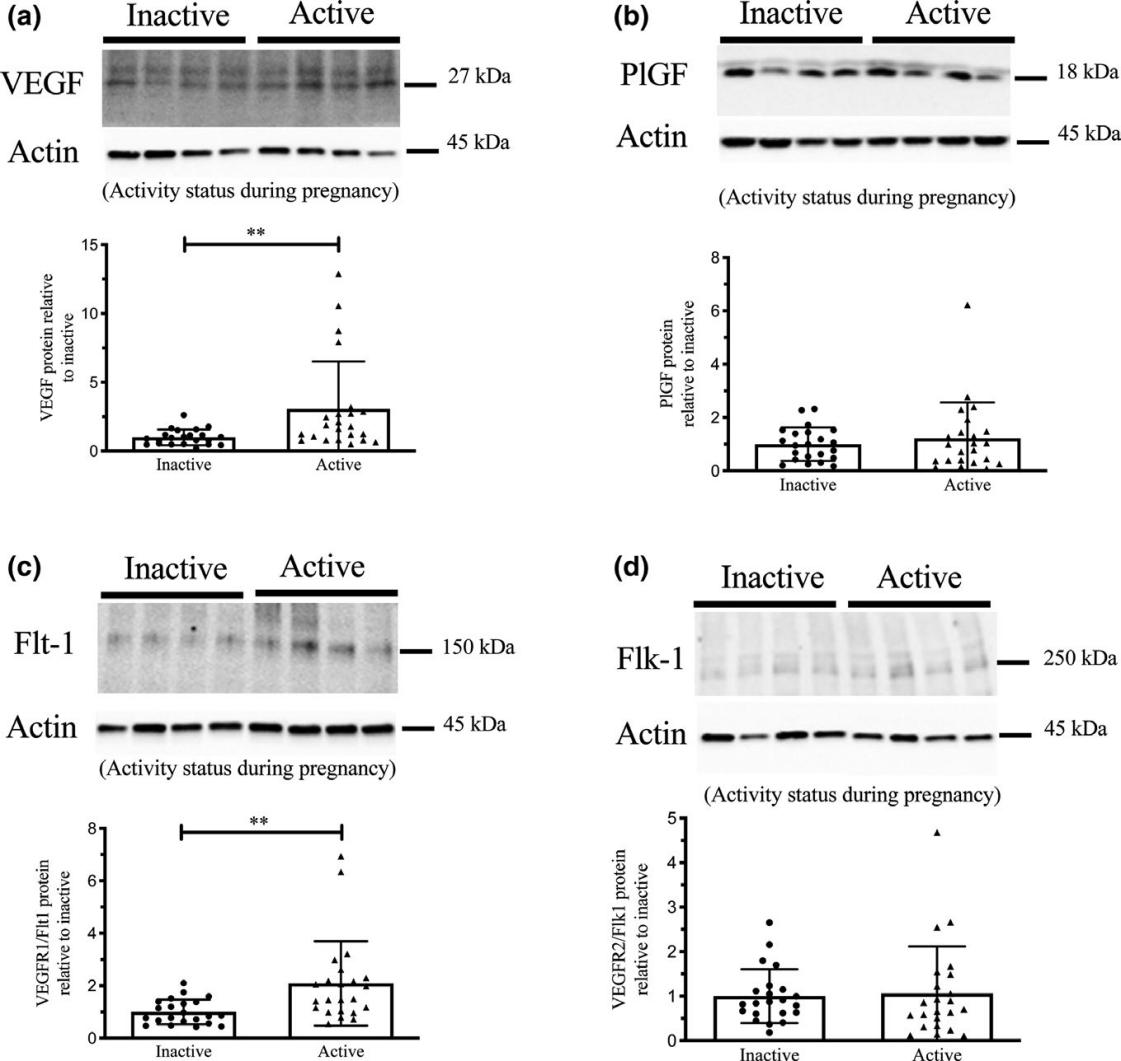
Expression of VEGF, PlGF, VEGFR1 (Flt1) and VEGFR2 (Flk1) protein by western immunoblotting in term placenta (2:1 ratio of tissue from the central and peripheral regions of the placenta, respectively) from physically active (*n* = 23) and inactive (*n* = 22) women. Representative immunoblots for VEGF (a, upper panel), PlGF (b, upper panel), VEGFR1 (Flt1) (c, upper panel), and VEGFR2 (Flk1) (d, upper panel) are shown. The corresponding semi‐quantitative densitometric analysis is shown for each protein in the lower panel of a–d, respectively. All data are represented as mean ± SD. **p < 0.01.

**TABLE 3:** Study participant demographics, exercise, and clinical information.

	Inactive (*n* = 22)	Active (*n* = 23)	*p*‐value
Maternal age (years)	31.5 ± 3.1	32.0 ± 3.0	0.556
Height (cm)	165.8 ± 7.1	166.5 ± 5.4	0.718
Pre‐pregnancy weight (kg)	66.4 ± 9.7	64.1 ± 11.3	0.525
Pre‐pregnancy BMI (kg/m^2^)	24.0 ± 3.6	23.3 ± 2.8	0.471
2nd trimester			
MVPA (min/day)	8.7 ± 5.2	43.6 ± 10.1****	<0.0001
MPA (min/day)	8.6 ± 5.2	36.4 ± 11.9****	<0.0001
VPA (min/day)	0.1 ± 0.5	7.2 ± 9.7****	<0.0001
LPA (min/day)	163.4 ± 46.0	179.9 ± 62.6	0.322
Steps per day	5083 ± 1515	9598 ± 2366****	<0.0001
Total caloric intake (kcal/day)^a^	2134 ± 419.3	2416 ± 551.1	0.087
CHO (g/day)	252.5 ± 50.2	315.4 ± 74.0**	0.004
Fiber (g/day)	21.4 ± 5.6	28.8 ± 8.0**	0.002
Sugars (g/day)	113.7 ± 37.4	137.3 ± 40.2	0.069
Protein (g/day)	91.6 ± 24.4	91.5 ± 21.7	0.989
Fats (g/day)	86.3 ± 22.4	93.4 ± 26.3	0.384
3rd trimester			
MVPA (min/day)	6.9 ± 5.9	23.2 ± 14.0***	0.0004
MPA (min/day)	6.8 ± 5.8	21.2 ± 12.0***	0.0003
VPA (min/day)	0.05 ± 0.1	2.0 ± 8.0	0.748
LPA (min/day)	175.1 ± 40.2	165.1 ± 46.3	0.528
Steps per day	5226 ± 2066	6693 ± 2085	0.054
Total caloric intake (kcal/day)^b^	2225 ± 488.6	2587 ± 571.8	0.104
CHO (g/day)	273.5 ± 73.9	312.7 ± 76.1	0.319
Fiber (g/day)	21.6 ± 5.0	27.9 ± 9.0**	0.003
Sugars (g/day)	120.4 ± 44.2	132.5 ± 41.0	0.401
Protein (g/day)	89.9 ± 18.7	102.9 ± 25.4	0.279
Fats (g/day)	87.7 ± 25.3	106.1 ± 29.8	0.054
Birth			
Gestational age at birth (weeks)	40.2 ± 1.2	40.0 ± 1.0	0.448
Mode of delivery	CS: 4 V: 18	CS: 3 V: 20	‐
Placental weight (g)	536.7 ± 97.7	472.9 ± 74.2*	0.017
Offspring sex	F: 11 M: 11	F: 12 M: 11	‐
Offspring birth weight (g)	3468 ± 392.7	3263 ± 329.9	0.092
Offspring birth length (cm)	51.0 ± 1.8	50.0 ± 2.2	0.095
Fetal: placental weight ratio (g/g)	6.6 ± 1.1	7.0 ± 0.9	0.240
Offspring birth weight percentile	42.8 ± 25.9	32.1 ± 22.0	0.116
Offspring body fat percentage (%)	17.52 ± 2.8	17.35 ± 2.7	0.830
Offspring weight‐for‐length z‐score	−0.35 ± 1.1	−0.25 ± 1.0	0.755

Values are shown as mean ± SD.

For 3rd trimester physical activity data, *n* = 21 active and *n* = 13 inactive, where group classifications are based on 2nd‐trimester physical activity.

BMI, body mass index; CS, cesarian section; F, female; M, male; CHO, carbohydrates; LPA, light intensity physical activity; MPA, moderate‐intensity physical activity; MVPA, moderate‐to‐vigorous‐intensity physical activity; VPA, vigorous‐intensity physical activity; V, vaginal.

^a^ Inactive *n* = 18 and active, *n* = 20.

^b^ Inactive *n* = 18 and active *n* = 18.

* *p* < 0.05,

** *p* < 0.01,

*** *p* < 0.001,

**** *p* < 0.0001.

### 3.2 VEGF, PlGF, VEGFR‐1 (FLT1), and VEGFR‐1 (FLK1) protein expression in placenta from physically active and inactive women

Figure 1a‐d represent the western blot analyses summarizing the expression of VEGF (~27 kDa), PlGF (~18 kDa), VEGFR‐1 (~150 kDa), and VEGFR‐2 (~250 kDa) in term placenta. Semiquantitative densitometric analysis showed the expression of VEGF and VEGFR‐1 were significantly (*p* < 0.01) higher in placenta from physically active compared to inactive women (Figure 1a and 1c, respectively). However, PlGF and VEGFR‐2 expression did not differ in placenta tissue from active versus inactive women (Figure 1b and 1d). Protein expression did not differ when comparing those who maintained “active” PA status into the 3rd trimester versus those who did not (i.e., active at 2nd and 3rd trimester vs. active at 2nd trimester only; data not shown). When stratified by offspring sex, there were no significant effects on protein expression (data not shown).

### 3.3 VEGF, PlGF, VEGFR‐1, and VEGFR‐2 mRNA expression in placenta from physically active and inactive women

**FIGURE 2:**
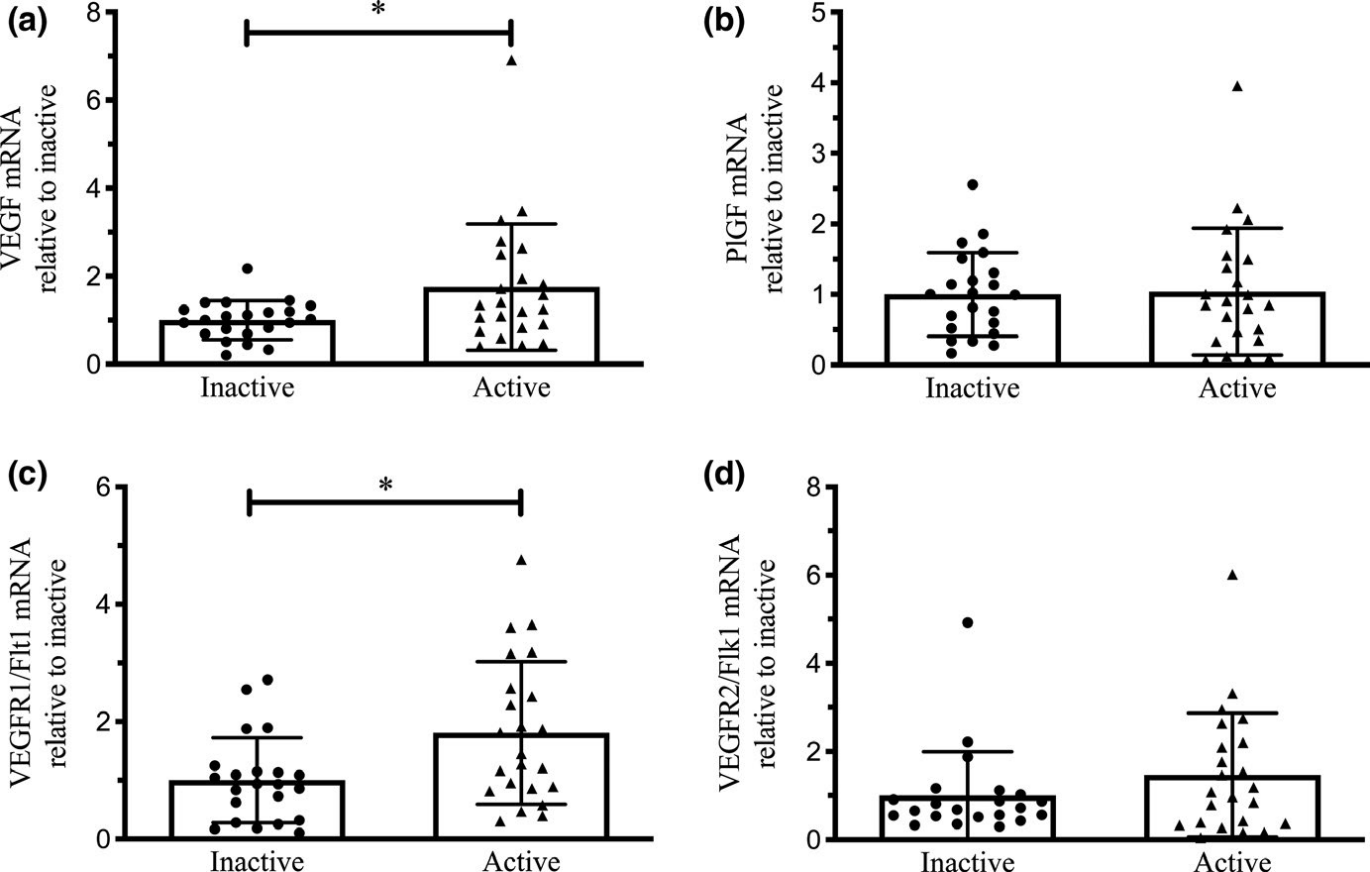
Expression of VEGF, PlGF, VEGFR1 (Flt1), and VEGFR2 (Flk1) mRNA by real‐time PCR in term placenta (2:1 ratio of tissue from the central and peripheral regions of the placenta, respectively) from physically active (*n* = 23) and inactive (*n* = 22) women. Relative quantification of mRNA VEGF (a), PlGF (b), VEGFR1 (Flt1) (c) and VEGFR2 (Flk1) (d) expression. Relative expression was normalized to the expression of GAPDH in all samples. All data are represented as mean ± SD. *p < 0.05.

The mRNA expression of *VEGF* and *VEGFR*‐*1* was higher in the placenta of active women (*p* < 0.05; Figure 2a,c). However, no differences between groups were found in *PlGF* and *VEGFR*‐*2* mRNA levels in placenta from either physically active or inactive women (Figure 2b,d). The expression of *VEGF*, *PlGF*, *VEGFR*‐*1*, and *VEGFR*‐*2* mRNA were not different when data were grouped by those who maintained an “active” PA status in 3rd trimester compared to those who did not meet activity status in 3rd trimester (i.e., only considered “active” at 2nd trimester; data not shown). No differences were found in any of the studied genes when data were categorized by offspring sex (data not shown).

### 3.4 Localization of VEGF, VEGFR‐1, and VEGFR‐2 in placenta from physically active and inactive women

**FIGURE 3:**
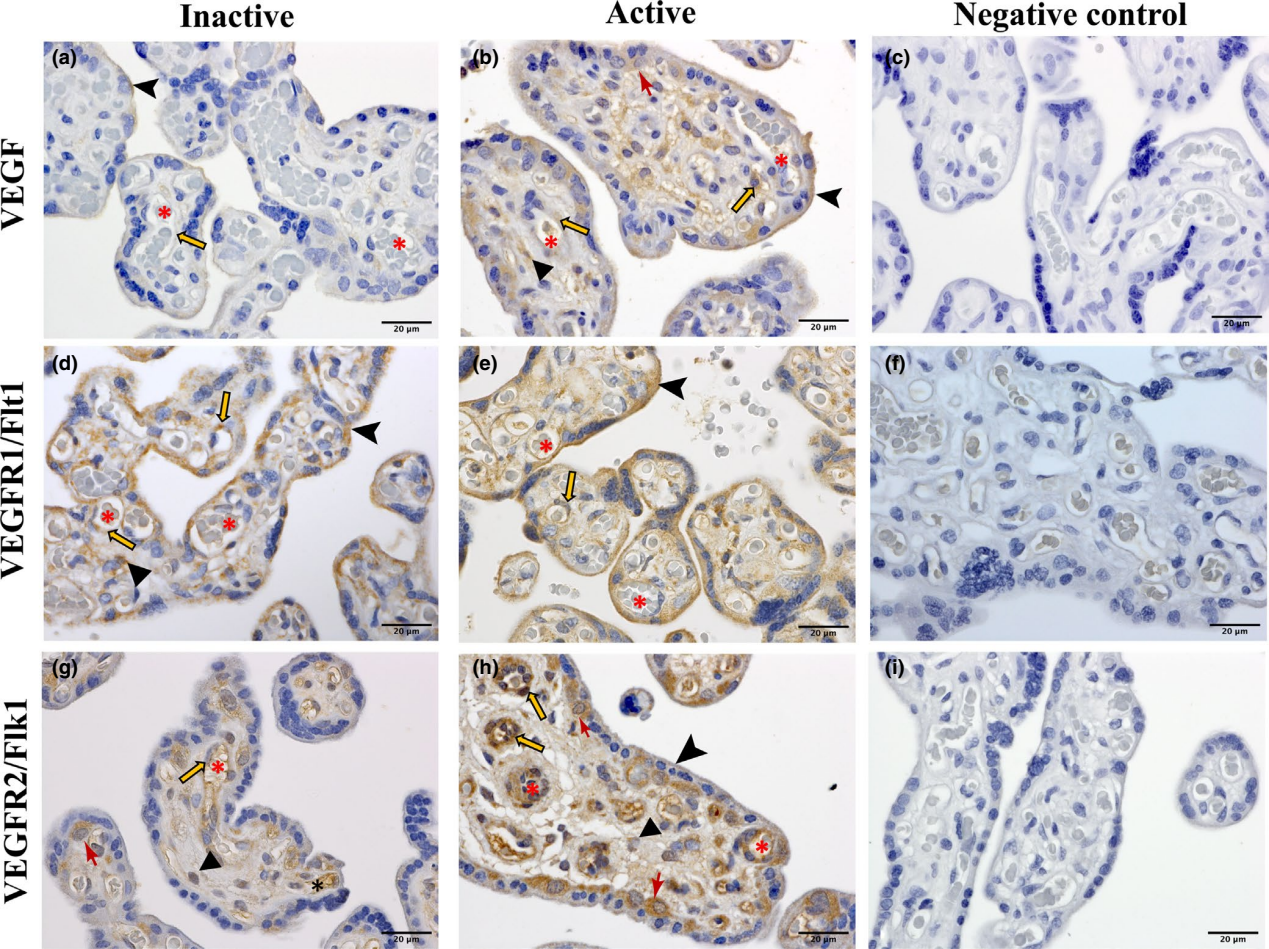
Immunolocalization VEGF (a, b), VEGFR1 (Flt1) (d, e), and VEGFR2 (Flk1) (g, h) in term placenta from physically active and inactive women. c, f, i represent negative controls, performed in absence of primary antibodies (diluent only). *red asterisk*, lumen of the blood vessels; *orange arrows*, endothelial cells; *triangles*, stromal cells; *arrow heads*, syncytiotrophoblast border, *red drafting point arrows*, cytotrophoblast cells. All sections were counterstained using hematoxylin. Scale bars, 20 µm.

Immunohistochemistry studies showed the localization of VEGF, VEGFR‐1, VEGFR‐2 in the placenta from both active and inactive women (Figure 3a–i). A distinctive VEGF staining pattern was noted in the placenta of active compared to inactive women (Figure 3b). In active women, intense VEGF staining was found in endothelial cells, stromal cells, syncytiotrophoblast border, and cytotrophoblast cells of the placenta (Figure 3b). However, in the placenta of inactive women, VEGF staining was observed mostly in the syncytiotrophoblast border, and relatively faint staining was detected in placental endothelial cells (Figure 3a) compared to the active group. Moreover, immunostaining of VEGF receptors, VEGFR‐1 and VEGFR‐2 were seen in endothelial cells, stromal cells, trophoblast cells of the placenta from both active and inactive women (Figure 3d,e,g,h). Importantly, intense staining of VEGFR‐2 was detected in both the endothelial and cytotrophoblast cells of the placenta from active (Figure 3h) compared to inactive women (Figure 3g). However, the immunostaining pattern of VEGFR‐1 was similar in endothelial, stromal, and trophoblast cells of both active and inactive placenta (Figure 3d,e). Negative control images are shown in Figure 3c,f,i. No reliable antibody for PlGF immunohistochemistry was available for study.

## 4 DISCUSSION

To the best of our knowledge, this is the first study showing the effect of PA during pregnancy on the VEGF pathway and its receptors in the human placenta. We observed higher VEGF expression at both the protein and mRNA levels in the placenta of physically active versus inactive women. We also report that PA increased the expression of VEGFR‐1, and did not affect the expression of VEGFR‐2 in the placenta of physically active women compared to the placenta of inactive women. While an earlier study showed higher VEGF expression in exercising pregnant rat placenta (Mangwiro et al., 2018), ours is the first in humans using a prospective, objective measurement of PA showing higher expression of VEGF and its receptor, VEGFR‐1 in the placenta of physically active women.

VEGF is expressed by endothelial and cytotrophoblast cells, in syncytiotrophoblast, and in stromal macrophages (Hofbauer cells) within the placental chorionic villi (Geva et al., 2002; Helske et al., 2001; Vuorela & Halmesmaki, 2006). Accordingly, we localized VEGF within endothelial, stromal, and trophoblast cells in the placenta from active and inactive women. We also found VEGF staining in the syncytial border of the chorionic villi. Distinct and differential staining of VEGF was evident in the placenta of active compared to inactive women. The placenta plays an important role in VEGF production, contributing to the proper vascular development of this critical organ (Ahmed et al., 2000; Burton et al., 2009). PA during pregnancy has been linked to increased levels of VEGF in circulation (Weissgerber et al., 2010). While we found increased VEGF in the placenta of women classified as “active” during pregnancy, the mechanisms remain unknown. Increased placental VEGF expression remained unaltered among women defined as “active” during the 2nd trimester and either maintained or reduced PA through to their 3rd trimester. While further investigation is required, this difference in expression indicates that habitual PA during the 2nd trimester might influence adaptative changes in the placenta. A meta‐analysis by Beetham et al. showed that even vigorous‐intensity PA during 3rd trimester is safe for most healthy pregnancies (Beetham et al., 2019). Likewise, PA into the 3rd trimester did not affect VEGF expression in the placenta. It is postulated that changes in arterial shear stress during exercise could be a contributing factor. Evidence from in vitro studies involving arterial shear stress on human umbilical vein endothelial cells (HUVECs) reported increased VEGF expression (dela Paz et al., 2012). Since the placenta lacks innervation, changes in shear stress due to enhanced blood flow during exercise in pregnancy could increase VEGF expression in endothelial and trophoblast cells in the placenta of active women.

Acute and chronic PA during pregnancy is theorized to create an intermittent hypoxic environment in the placenta due to the redistribution of maternal blood and oxygen within systemic circulation (Clapp, 2003; Clapp et al., 2000). However, this phenomenon remains unconfirmed in human pregnancy in the context of either acute or habitual moderate‐intensity PA. Hypoxia has been found to upregulate VEGF expression and its receptors in human myeloma cells and in human placental choriocarcinoma cells (BeWo) (Giatromanolaki et al., 2010; Trollmann et al., 2003). Hence, increased VEGF expression in the placenta of active women could also result from hypoxia‐mediated events. The functional role or consequence of increased VEGF, at both the protein and gene level, in the placenta of active women is still unexplored. Our current findings suggest that increased VEGF expression may support vascular adaptations by potentially increasing placental branching vasculogenesis, ultimately improving placental function by modulating its nutrient transfer. In early placental vascularization and development, lower VEGF levels have been reported to be related to the development of disorders known to have affected vascularization, including preeclampsia and/or intrauterine growth restriction (Dymara‐Konopka et al., 2018; Xu et al., 2016). Moreover, it has been found that maternal caruncle and fetal cotyledonary expression of VEGF and its receptors are positively correlated with placental vascularization and uteroplacental, and fetal blood flow in the pregnant ewe (Borowicz et al., 2007; Zheng et al., 1997). Although we did not confirm changes in the structural morphology of the placental vascular network, previous studies have reported enhanced perfusion and improved vascularization due to PA in pregnancy (Jackson, Clapp 2000 Beginning, Bergmann, 2004). Therefore, our findings may implicate PA as a potential therapeutic target for the optimization of placental health and function.

Earlier research in humans showed that regular PA throughout pregnancy (minimum 3 h/week) increased plasma PlGF levels measured in late gestation (Weissgerber et al., 2010). This systemic increase of PlGF could be due to its release from either the placenta or other organs. Still, no data are available regarding the expression of PlGF in the placenta from active and inactive women. Research from a rat model of PA during pregnancy showed decreased PlGF protein expression in the placenta of male offspring, whereas no changes were observed in the placenta of female offspring (Mangwiro et al., 2018). Our study did not find any differences in PlGF protein and mRNA expression level in placenta tissue from active and inactive women. We also did not find any differences between groups when we stratified by offspring sex. As pregnancy progresses, oxygen tension increases with concomitant increases in PlGF expression in the term placenta. Similarly, *in vitro* work using a term placental choriocarcinoma cell line (BeWo cells) found that increasing oxygen tension increased PlGF expression (Ahmed et al., 2000). Conversely, hypoxia has been found to downregulate PlGF in vitro in BeWo cells (Ahmed et al., 2000). In our study, despite presumed PA‐mediated intermittent placental hypoxia in women categorized as active, no change in PlGF expression was evident in the placenta of active compared to inactive women. Physiologically relevant levels of PlGF have been illustrated to be a weak stimulator of endothelial cell chemotaxis and proliferation (Birkenhager et al., 1996). Of interest, murine studies have shown that PlGF is not required for PA‐induced angiogenesis in the heart and skeletal muscle (Gigante et al., 2004).

In the process of angiogenesis and vasculogenesis, both VEGF and PlGF elicit their effects via binding to their receptors, VEGFR‐1 and VEGFR‐2. PlGF may potentiate the action of VEGF by binding with VEGFR‐1, thus allowing for more VEGFR‐2 to bind with VEGF (Park et al., 1994) in the placenta. PlGF generally mediates non‐branching angiogenesis by binding with VEGFR‐1 and inducing endothelial tube formation (Fong et al., 1995; Kurz et al., 1998). Increased VEGF expression without changes in the level of PlGF might indicate that VEGF‐mediated branching angiogenesis is greater in the placenta of active women than PlGF‐mediated non‐branching angiogenesis, which requires further investigation.

We observed increased expression of VEGFR‐1 and no changes in VEGFR‐2 in the placenta of active women at the mRNA and protein levels. Earlier maternal exercise studies in rats showed no differences in placental VEGFR‐1 expression in the case of male offspring; however, a decrease was observed in female offspring exposed to a high‐fat diet (Mangwiro et al., 2018). We determined that VEGFR‐2, known for its pro‐angiogenic activity, was similar in the placenta of both physically active and inactive women. Analysis by offspring sex did not reveal any differences between groups. Localization by immunostaining did not show any striking differences in VEGFR‐1 between the placenta from active and inactive women. However, marked endothelial and trophoblast staining of VEGFR‐2 was evident in the placenta of active women. Both VEGFR‐1 and VEGFR‐2 were found in endothelial, stromal, and trophoblast cells of the placenta from active and inactive women, consistent with previous studies (Demir et al., 2004). Also, PA has been found to increase VEGFR‐1 and VEGFR‐2 in rat skeletal muscle and in mouse heart (Gavin & Wagner, 2002; Lloyd et al., 2003). In the placenta, exercise‐induced shear stress could explain the upregulation of VEGF receptors as described previously in endothelial progenitor cells and in embryonic stem cells (Kutikhin et al., 2018; Yamamoto et al., 2005). The impact of shear stress in the placenta and its relationship with angiogenesis has yet to be observed. As PlGF only binds to VEGFR‐1 and not with VEGFR‐2, increased VEGFR‐1 may act as a predominant binding site for PlGF in the placenta of active women. Occasionally, the binding of VEGF with VEGFR‐1 may also be pro‐angiogenic as previous studies have documented VEGF/VEGFR‐1 interaction in endothelial tube formation (Fong et al., 1995, 1996).

We observed reduced placental weight in physically active compared to inactive women, with no difference in fetal weight. We found that offspring weight and length were lower in the active group, albeit not statistically significant but may account for a smaller placenta. The association between PA during pregnancy and placental weight is inconsistent within the literature (Clapp & Capeless, 1990; Ramirez‐Velez et al., 2013). However, we did not observe a difference in placental:fetal weight ratio, a surrogate marker of placental efficiency in line with recent work from our lab, and others (Everest et al., 2020; Hilde et al., 2017; Juhl et al., 2010). A study with a larger population is obligatory to validate the effect of exercise on placental weight.

It must be noted that our current study focused on the examination of key angiogenic mediators in the term placenta when the growth of its vasculature has peaked. Due to the nature of research involving pregnant women, and the lack of non‐invasive methods available for studying the human placenta, it is only possible to examine term placenta tissue to gain insight into the molecular mechanisms underlying healthful pregnancies. We must also consider the timing of PA measurement as a limitation. Activity status was determined based on the measurement of PA by accelerometry during the 2nd trimester assessment period. We chose to define activity status during this study window because it coincides with the critical phase of placental growth and development. The 2nd trimester is also a time during pregnancy when a mother is most comfortable engaging in exercise and is most likely to volunteer for research studies. Our study presents some important strengths; namely, the objective measurement of PA in pregnant women using a larger sample size than has been examined in the literature to‐date. Furthermore, we accounted for potential confounding factors, including dietary intake and pre‐pregnancy BMI. All maternal and neonatal anthropometric measurements were conducted by the researchers in a consistent and systematic manner. We recognize that pre‐pregnancy PA data could strengthen the study, but considering that a retrospective self‐report questionnaire would be the only way to collect this data, we opted not to rely on recalled PA habits before pregnancy. Although great care was taken to ensure precise data measurement, we acknowledge that proof‐of‐concept studies using appropriate *in vitro* cell models should be explored to assess the relationship between PA and placental angiogenesis. That being said, this study is the first of its kind to link objectively measured PA metrics in healthy pregnancies to placental angiogenesis.

Our findings of increased VEGF, VEGFR‐1 expression, and distinct VEGFR‐2 staining in the endothelial cells of the placenta of active women suggest that placental VEGF binding may lead to enhanced pro‐angiogenic outcomes. Overall, our study shows that PA may promote angiogenesis by increasing VEGF expression.

## CONFLICTS OF INTEREST

No conflicts of interest declared by the authors.

## AUTHOR CONTRIBUTION

JB drafted the manuscript. JB and SM primarily performed data collection and analysis. AG secondarily performed data entry. All authors designed the study with KBA as the leading author. All authors revised, edited, and approved the final version of the manuscript.

## Supporting information



Fig S1

## Data Availability

The data that support the findings of this study are available from the corresponding author, upon reasonable request.
