# Supplementary material for: Physical activity differentially regulates VEGF, PlGF, and their receptors in the human placenta
Source: Physiol Rep. 2021 Jan 19;9(2):e14710. doi: 10.14814/phy2.14710 (PMC7814495; doi:10.14814/phy2.14710)
Supplement: Supplementary file 1 [file PHY2-9-e14710-s001.docx]

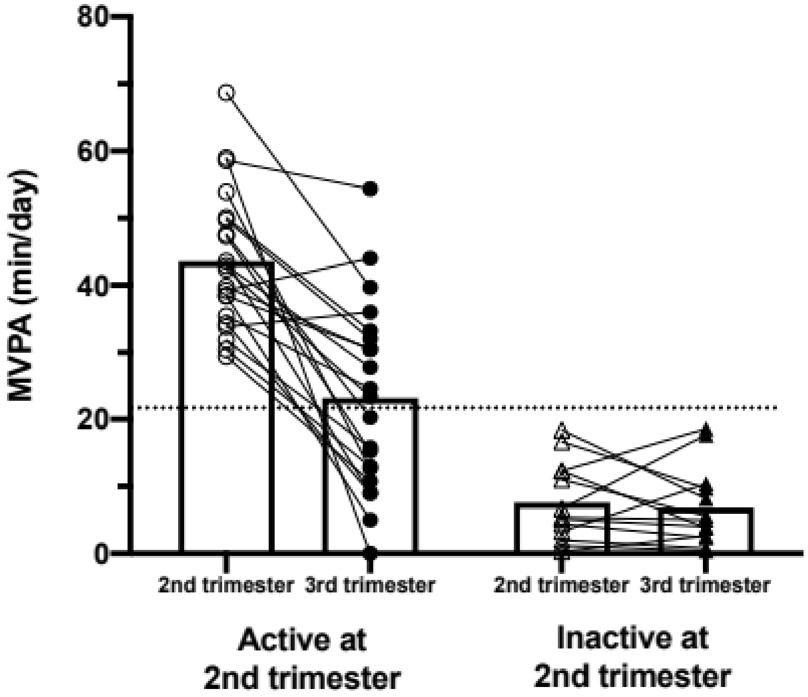
MVPA (min/day)
80
60
40
20
0
2nd trimester
3rd trimester
2nd trimester
3rd trimester
Active at
2nd trimester
Inactive at
2nd trimester
